# Supplementary material for: Genomic epidemiology and multilevel genome typing of Bordetella pertussis
Source: Emerg Microbes Infect. 2023 Aug 2;12(2):2239945. doi: 10.1080/22221751.2023.2239945 (PMC10399484; doi:10.1080/22221751.2023.2239945)
Supplement: Supplemental Material [file TEMI_A_2239945_SM8039.pdf]

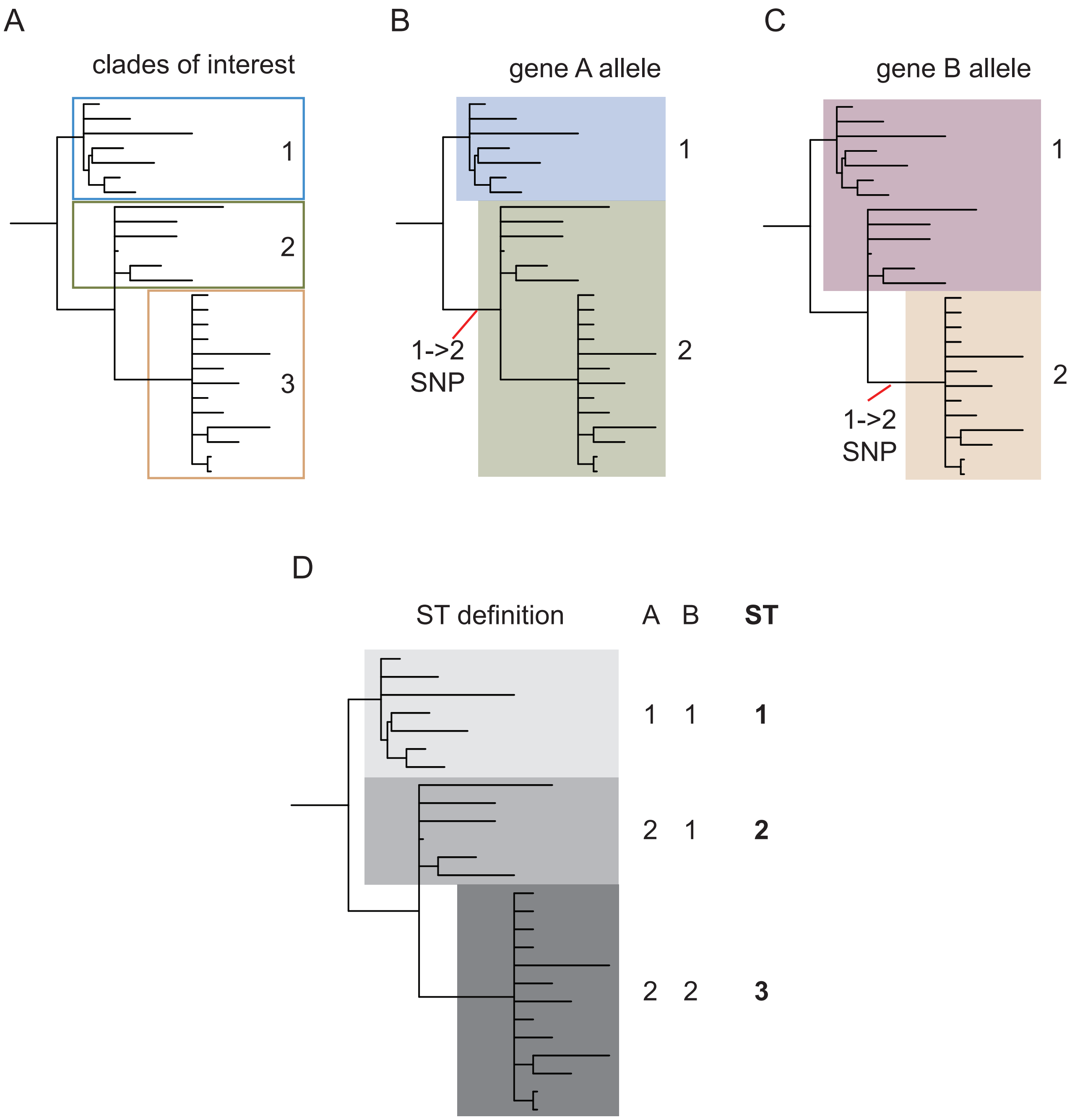

Supplementary figure 1. Phylogenetically informative loci selection for MGT2 and MGT3. **A.** An example phylogeny showing three clades of interest. **B.** Gene A has a SNP on the ancestral branch of clades 2 and 3 that leads to geneA having one allele for clade 1 and another for clades 2 and 3. **C.** A similar example to A with gene B alleles distinguishing clade 3 from clades 1 and 2. **D.** Combination of alleles in gene A and gene B into an allele profile defines three distinct STs that act as a nomenclature for the clades of interest.

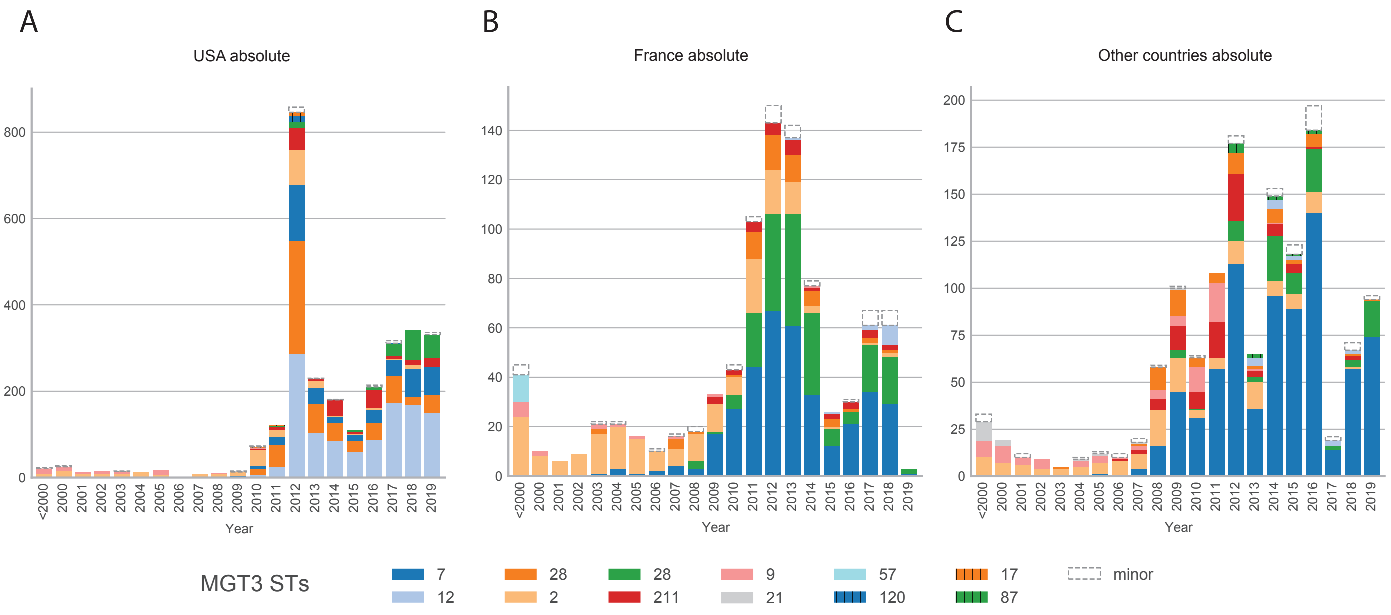

Supplementary Figure 2. Temporal changes in MGT2-MGT3 ST counts. The count of isolates assigned to each major MGT3 ST within MGT2 ST2 in each year are shown by different colours in each column. **A.** Counts of STs in the USA over time. **B.** Counts of STs in the France over time. **C.** Counts of STs in the countries other than the USA and France over time. Isolates assigned to minor STs within MGT2 ST2 are grouped together and are indicated by a dashed outline.

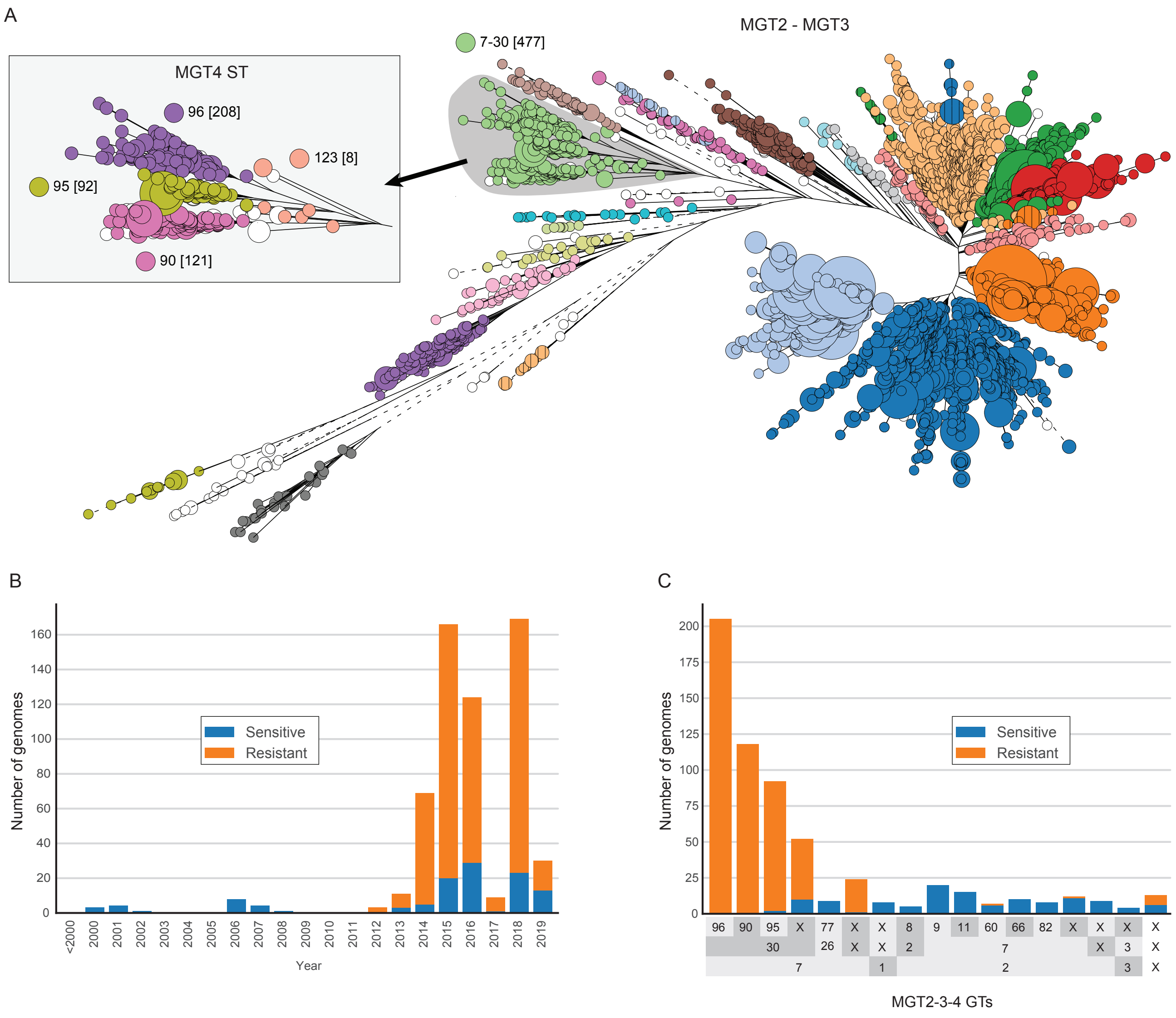

Supplementary Figure 3. Description of three erythromycin resistant lineages from China. A. A neighbour joining phylogeny generated from MGT5 allele profiles. Nodes are coloured by MGT2 and MGT3 STs as indicated by labels (identical to figure 2). The inset shows an expanded view of the MGT3 ST30 clade coloured by MGT4 ST. The three resistant lineages are MGT4 ST90, MGT4 ST95 and MGT4 ST96. **B.** All genome sequences available from China up to 2019 grouped by year and coloured by predicted erythromycin resistance. **C.** All genome sequences available from China with assigned MGT2, MGT3 and MGT4 STs on the X-axis. Predicted erythromycin resistance shown by colour. Columns with the same MGT2 and/or MGT3 STs are grouped together. Isolates from STs containing fewer than 6 isolates were collapsed into a single type and labelled X. e.g. 7-X-X isolates were assigned to MGT2 ST7 but their MGT 3 and MGT4 STs contained fewer than 6 isolates.

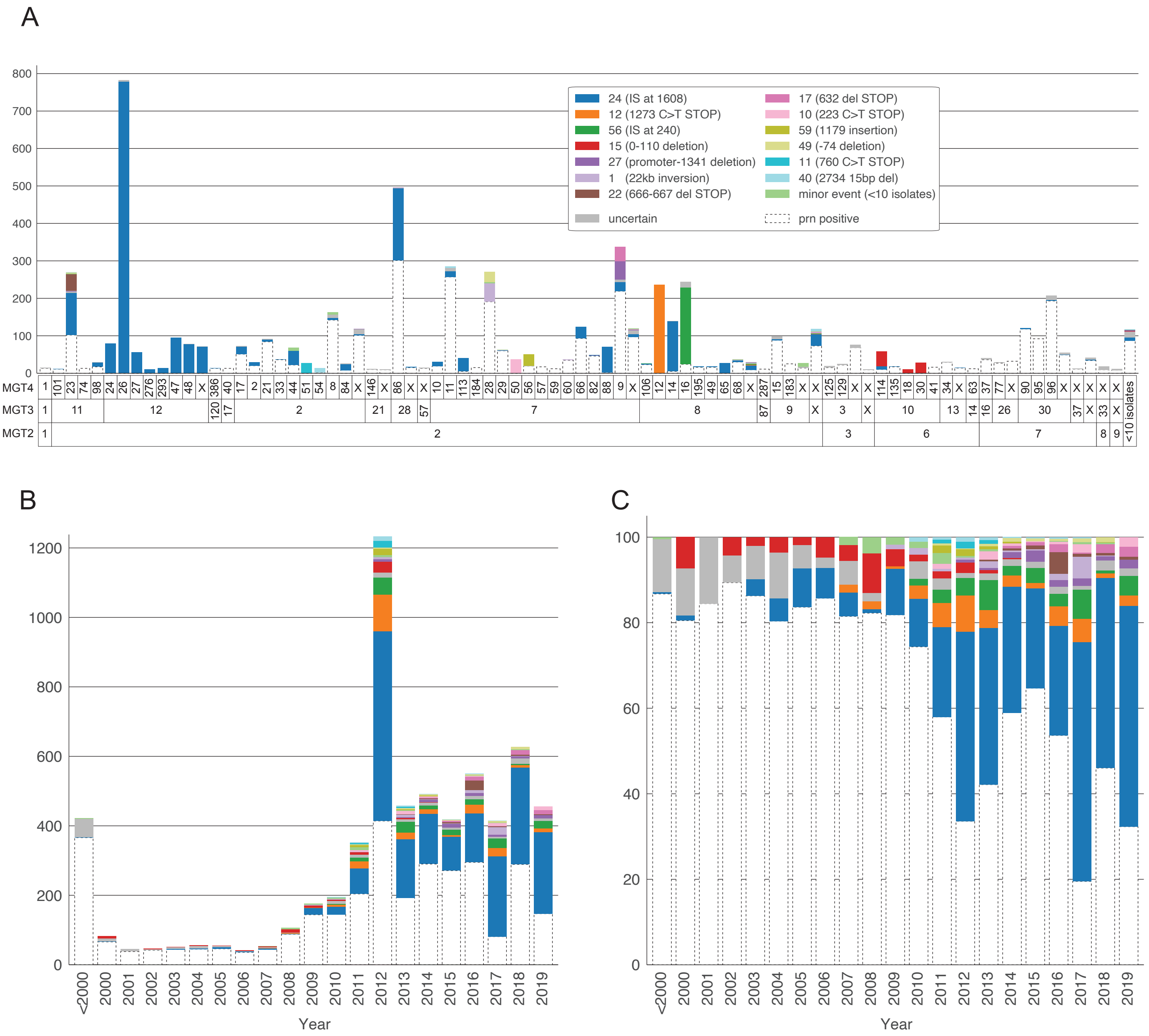

Supplementary Figure 4. Distributions of prn disruption causes across MGT types and years. A. Each column is unique set of MGT2, MGT3 and MGT4 STs with 10 or more isolates assigned to it. Each columnis coloured by prn disruption type (colours are the same for all three graphs). prn positive isolates are marker with white dashed outline. The same MGT3 and MGT2 STs are grouped together to allow interpretation at MGT2, MGT3 and MGT4 levels. Isolates from STs containing fewer than 6 isolates were collapsed into a single type and labelled X. e.g. 9-X-X isolates were assigned to MGT2 ST9 but their MGT 3 and MGT4 STs contained fewer than 6 isolates. **B.** The absolute numbers of isolates that are assigned to each disruption type in each year. **C.** The proportion of isolates that are assigned to each disruption type in each year.

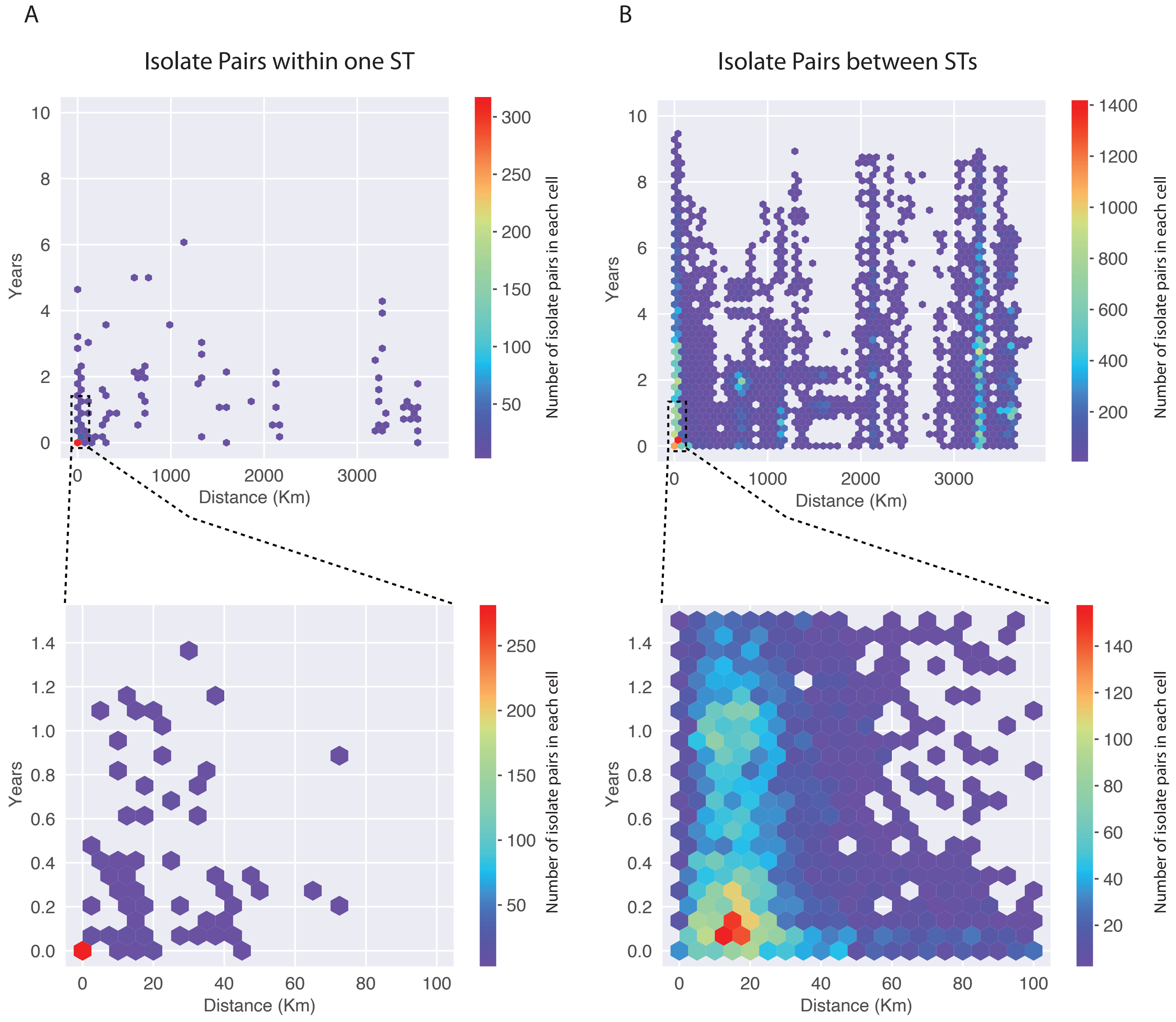

Supplementary figure 5. Temporal and spatial distributions of isolate pairs within or between MGT5 STs. Distribution of pairwise distances by year (y axis) and by kilometers (x axis). Data are grouped into cells where the number of isolate pairs falling into each is indicated by its colour. A. Isolate pairs that are both assigned the same MGT5 ST. B. Isolate pairs that are assigned different MGT5 STs. Zoomed in distribution of 0-1.5 years and 0-100km are shown beneith each main plot.
